# Editorial: Modern Statistical Learning Strategies in Imaging Genetics

**DOI:** 10.3389/fnins.2022.957304

**Published:** 2022-06-15

**Authors:** Chao Huang, Rongjie Liu, Bingxin Zhao, Linglong Kong

**Affiliations:** ^1^Department of Statistics, Florida State University, Tallahassee, FL, United States; ^2^Department of Statistics, Purdue University, West Lafayette, IN, United States; ^3^Department of Mathematical and Statistical Sciences, University of Alberta, Edmonton, AB, Canada

**Keywords:** imaging genetics, causal inference, deep learning, high dimensionality, mediation analysis

With the rapid growth of modern technology, many biomedical studies, such as the Alzheimer's disease neuroimaging initiative (ADNI) study (Mueller et al., 2005), Human Connectome Project (HCP) (Van Essen et al., 2013), and UK BioBank (UKBB) study (Sudlow et al., 2015), are being conducted to collect massive datasets with volumes of multi-modality imaging, genetic, neurocognitive, and clinical information from increasingly large cohorts. Integrating these rich and diverse heterogeneous information can help our understandings of how genetic variants impact brain structure, brain function, cognition, and brain-related disease risk across the lifespan. However, the development of statistical learning methods in imaging genetics presents significant computational and theoretical challenges caused by the high-dimensional nature of both imaging phenotypes and genetic data. Meanwhile, existing analytical methods also face challenges in characterizing the spatial dependence in various neuroimaging measures and dependence structures in genetic markers from linkage disequilibrium. In addition, a long-term challenge in the imaging genetics field is the limited sample size of traditional imaging studies, which may have low power in detecting the polygenic genetic architecture of brain diseases and cause overfitting of statistical learning models. This special issue includes a group of papers specifically leveraging these massive biomedical datasets to developing new learning approaches in imaging genetics and uncover novel clinical findings.

Matrix decomposition and low-rank representation techniques have been seen as powerful tools in handling the high-dimensionality issue in brain image data and detecting imaging biomarkers for the diagnosis of mental disorders. Tu et al. developed a low-rank plus sparse matrix decomposition technique to construct the connectivity matrix for the functional magnetic resonance imaging (fMRI) data. They applied the proposed pipeline to resting-state fMRI data in ADNI study and showed that the new method increased the detectability of group differences for Alzheimer's disease (AD). Wu et al. introduced a co-sparse non-negative matrix factorization method to high-dimensional brain image data by simultaneously imposing sparsity in both two decomposed matrices. They found that the proposed method successfully detected difference between AD patients and normal person in several brain regions when applying to two datasets, i.e., structure MRI and fMRI, in ADNI study. In addition, the high-dimensionality issue is also ubiquitous in genetic data. Ridge-penalized tests for high-dimensional hypothesis testing problems were investigated in Gauran et al., and a class of methods for choosing the optimal ridge penalty were developed as well. They proposed strategies to improve the statistical power of ridge-penalized tests and applied them to an imaging genetics study where the associations between a set of candidate single nucleotide polymorphisms (SNPs) and the electroencephalogram (EEG) coherence were tested.

As an alternative to association analysis, causal inference has attracted more and more attention for the discovery of mechanical relationships among imaging phenotypes, genetic exposures, and clinical outcomes. Chen et al. focused on the problem how genetic architecture and brain connectome causally affect human behaviors in the HCP study. They carried out the causal pathway analysis from single nucleotide polymorphism (SNP) data to four common human cognitive traits, mediated by the brain connectome. They found that a majority of the selected SNPs have significant direct effects on human traits and indirect effects through trait-specific brain connectomes. Ye et al. hypothesized that the vertical pleiotropic pathways, where genetic variants influence a trait that in turn influences another trait, link genetic factors, integrity of cerebral white matter (WM), and nicotine addiction. They tested this hypothesis using individual genetic factors, WM integrity measured by fractional anisotropy, and nicotine dependence-related smoking phenotypes, in the UKBB study. Their causal pathway analysis revealed the role of cerebral WM in the maintenance of the complex addiction and provided potential genetic targets for future research in examining how changes in WM integrity contribute to the nicotine effects on the brain. Ghosh et al. developed an inferential framework for estimating causal effects with radiomics data. They leveraged a multivariate version of partial least squares for causal effect estimation, which addressed the challenge that the exposure of interest is latent. The proposed methodology was demonstrated through two applications on the radiomics datasets, one in osteosarcoma and the other one in glioblastoma.

Finally, integration of imaging and genetic data through deep learning techniques recently gained considerable attention in AD prediction. Wang et al. developed a deep learning approach, named IGnet, for automated AD classification using both MRI and genetic data. They applied the proposed approach to the baseline MRI scans and selected SNPs on chromosome 19 in ADNI study, which achieved a classification accuracy of 83.78% and an area under the receiver operating characteristic curve (AUC-ROC) of 92.4% in the test set.

Taken together, the studies in this special issue include several advanced statistical learning approaches in imaging genetics, and exemplify the potential impact of applying these methods to better understand the roles of brain imaging data and genetic information in mental health and disease.

## Author Contributions

CH and RL wrote the editorial. BZ and LK contributed to manuscript polishing. All authors contributed to the article and approved the submitted version.

## Conflict of Interest

The authors declare that the research was conducted in the absence of any commercial or financial relationships that could be construed as a potential conflict of interest.

## Publisher's Note

All claims expressed in this article are solely those of the authors and do not necessarily represent those of their affiliated organizations, or those of the publisher, the editors and the reviewers. Any product that may be evaluated in this article, or claim that may be made by its manufacturer, is not guaranteed or endorsed by the publisher.
